# Supplementary material for: A Novel SLPI Splice Variant Confers Susceptibility to Otitis Media in Humans
Source: Int J Mol Sci. 2025 Feb 7;26(4):1411. doi: 10.3390/ijms26041411 (PMC11855725; doi:10.3390/ijms26041411)
Supplement: Supplementary file 1 [file ijms-26-01411-s001.zip › ijms-3425346-supplementary.pdf]

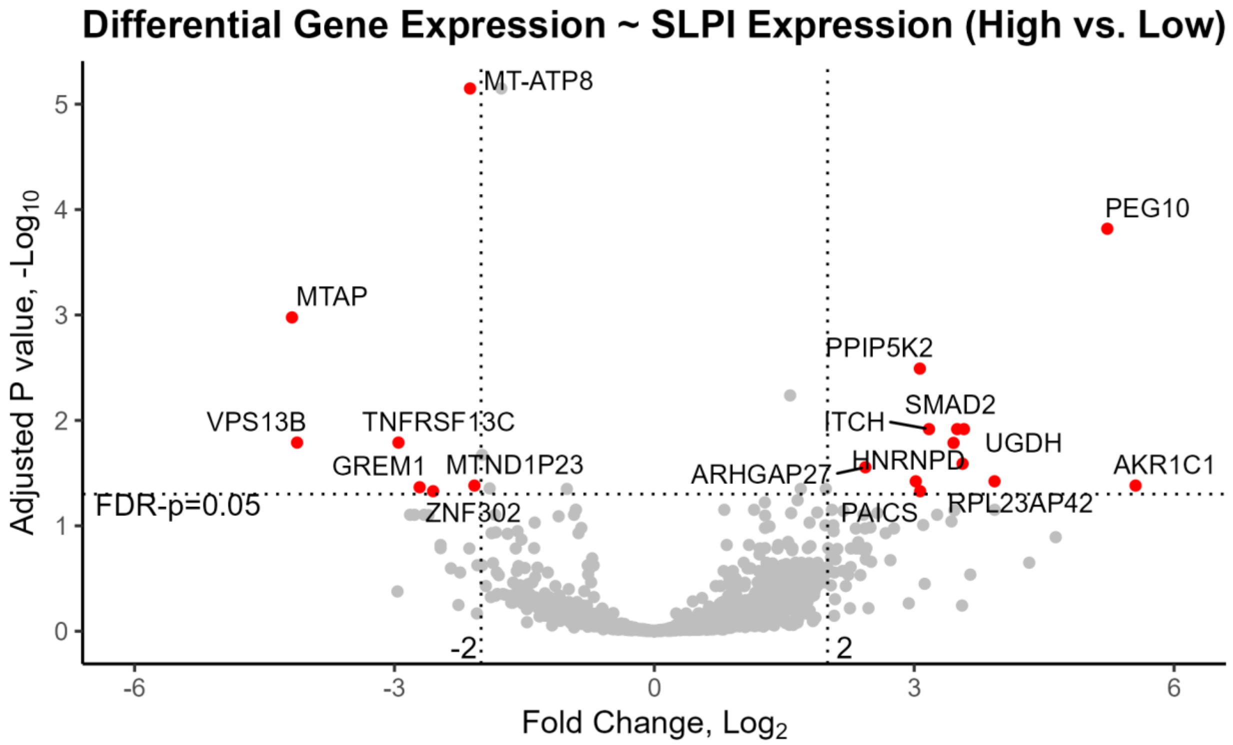

Figure S1. Volcano plot of genes that are differentially expressed between high- vs low-expressers of *SLPI*. Two genes *SURF4* and *MGLL* were also significant in the DESeq2 analysis but are not labeled in the volcano plot.

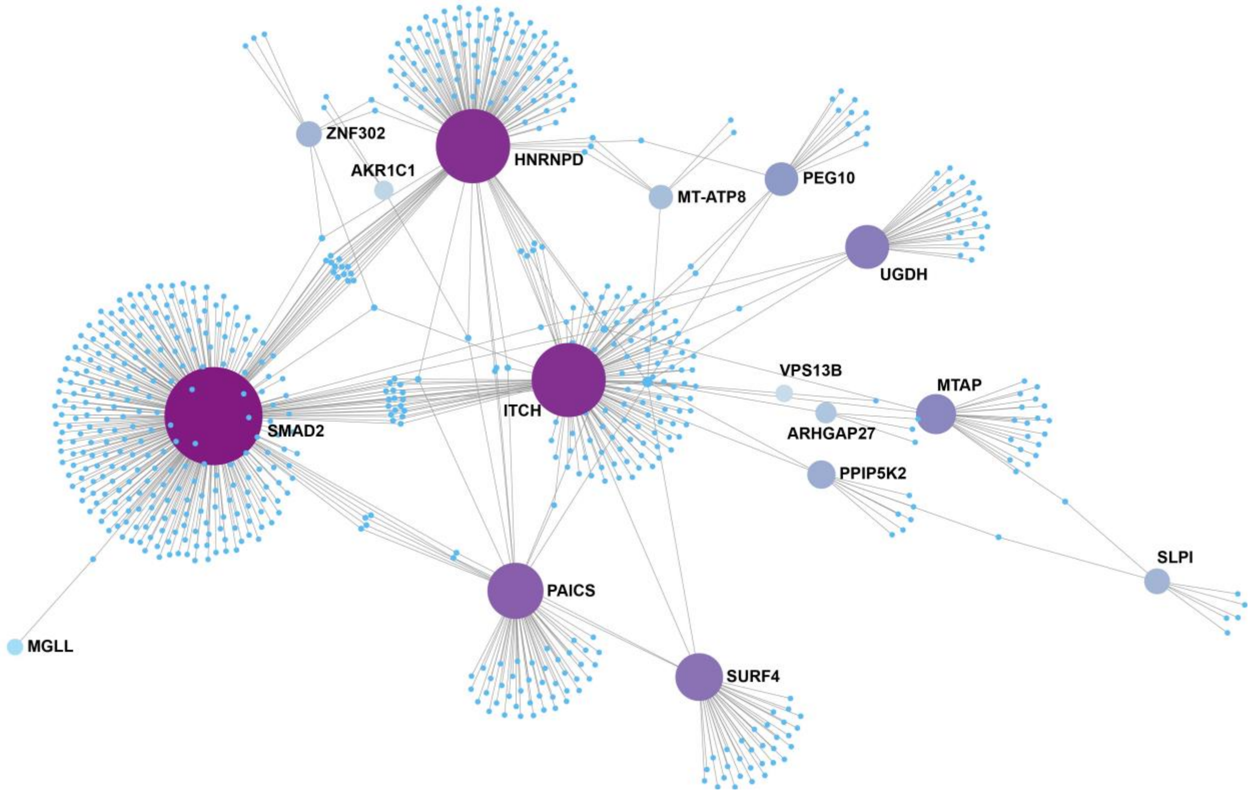

Figure S2. Network of differentially expressed genes. Fifteen of the nineteen differentially expressed genes (Figure S1) and *SLPI* are connected by a single interaction network.

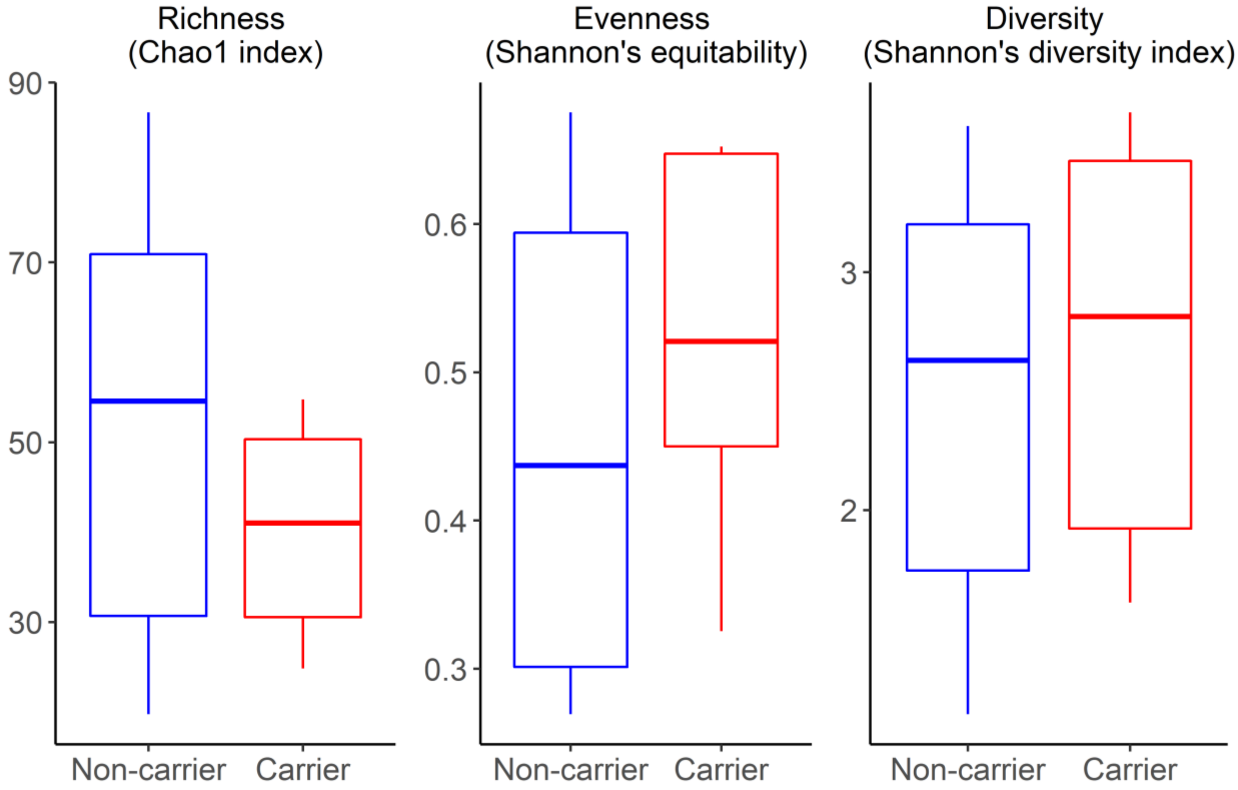

Figure S3. Alpha-diversity indices in the middle ear by *SLPI* c.394+1G>T variant carriage. Alpha-diversity indices indicate no significant differences based on variant carriage.

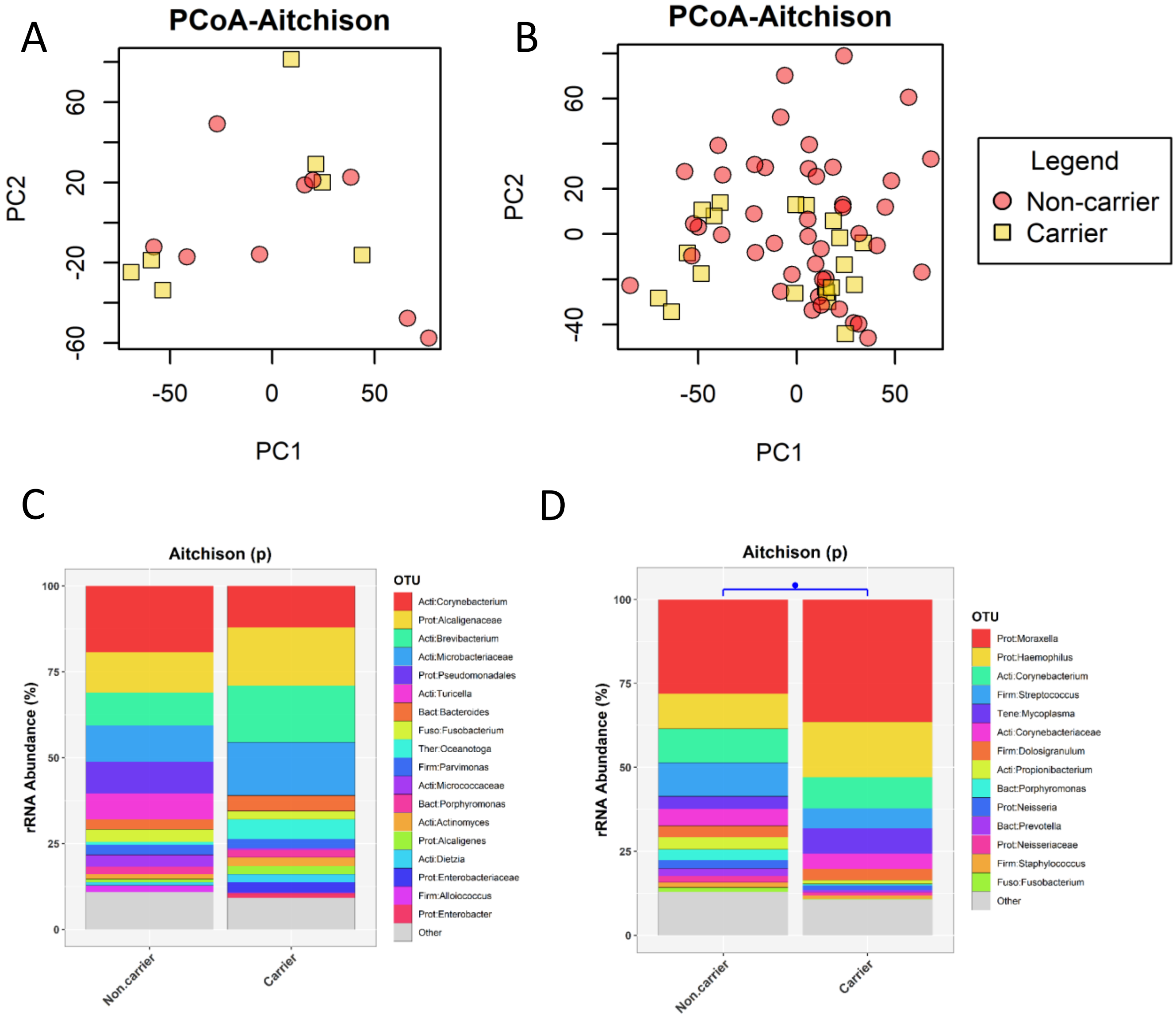

Figure S4. Beta-diversity by *SLPI* c.394+1G>T variant carriage. The first two panels show the principal coordinate analysis (PCoA) by Aitchison distance in the (A) middle ears and (B) nasopharynges. (C) Cumulative relative abundance profiles of the middle ears of *SLPI* c.394+1G>T variant carriers and non-carriers. Beta-diversity between the two groups as measured by Aitchison distance was not significant. (D) Cumulative relative abundance profiles of the nasopharynges of *SLPI* c.394+1G>T variant carriers and non-carriers. Beta-diversity between the two groups as measured by Aitchison distance is only nominally significant (nominal-p=0.04) after adjusting for sequencing batch (p=1x10^{-6}) and age (p=0.67).

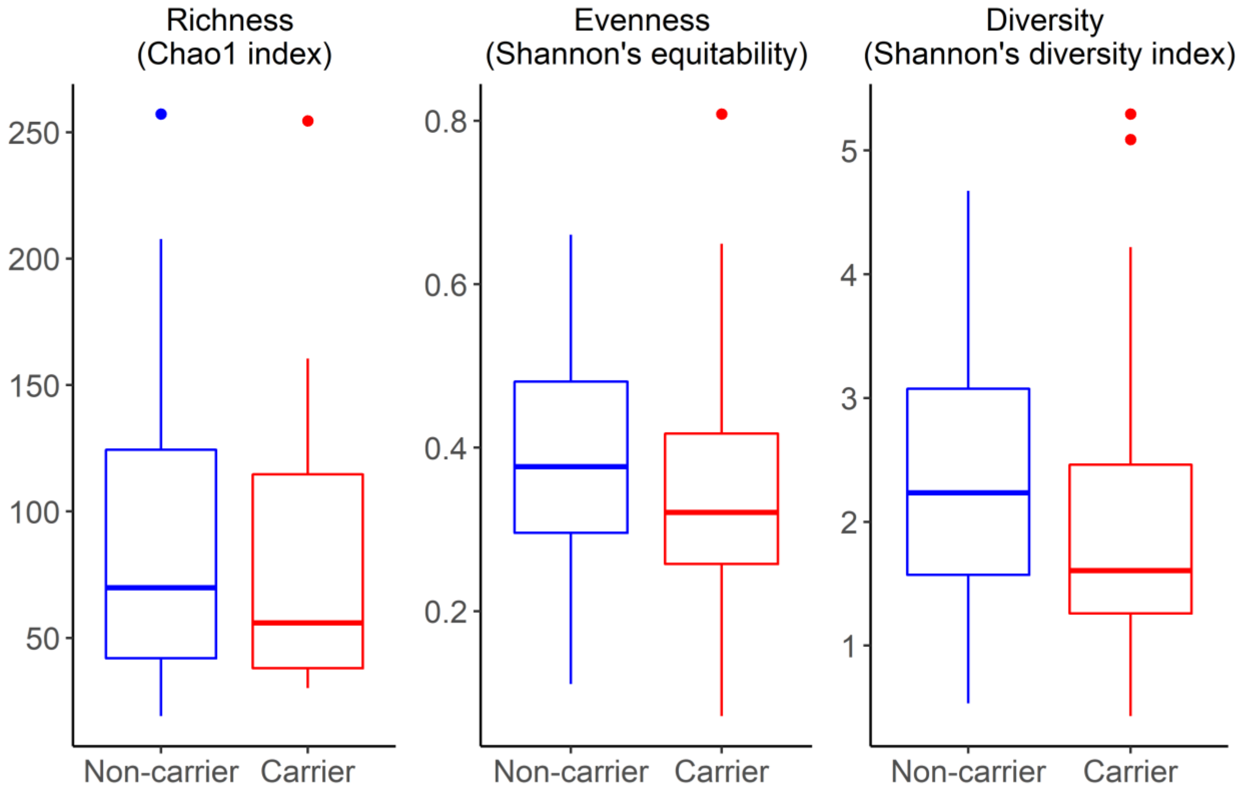

Figure S5. Alpha-diversity indices in the nasopharynx by *SLPI* c.394+1G>T variant carriage. Alpha-diversity indices indicate no significant differences based on variant carriage.

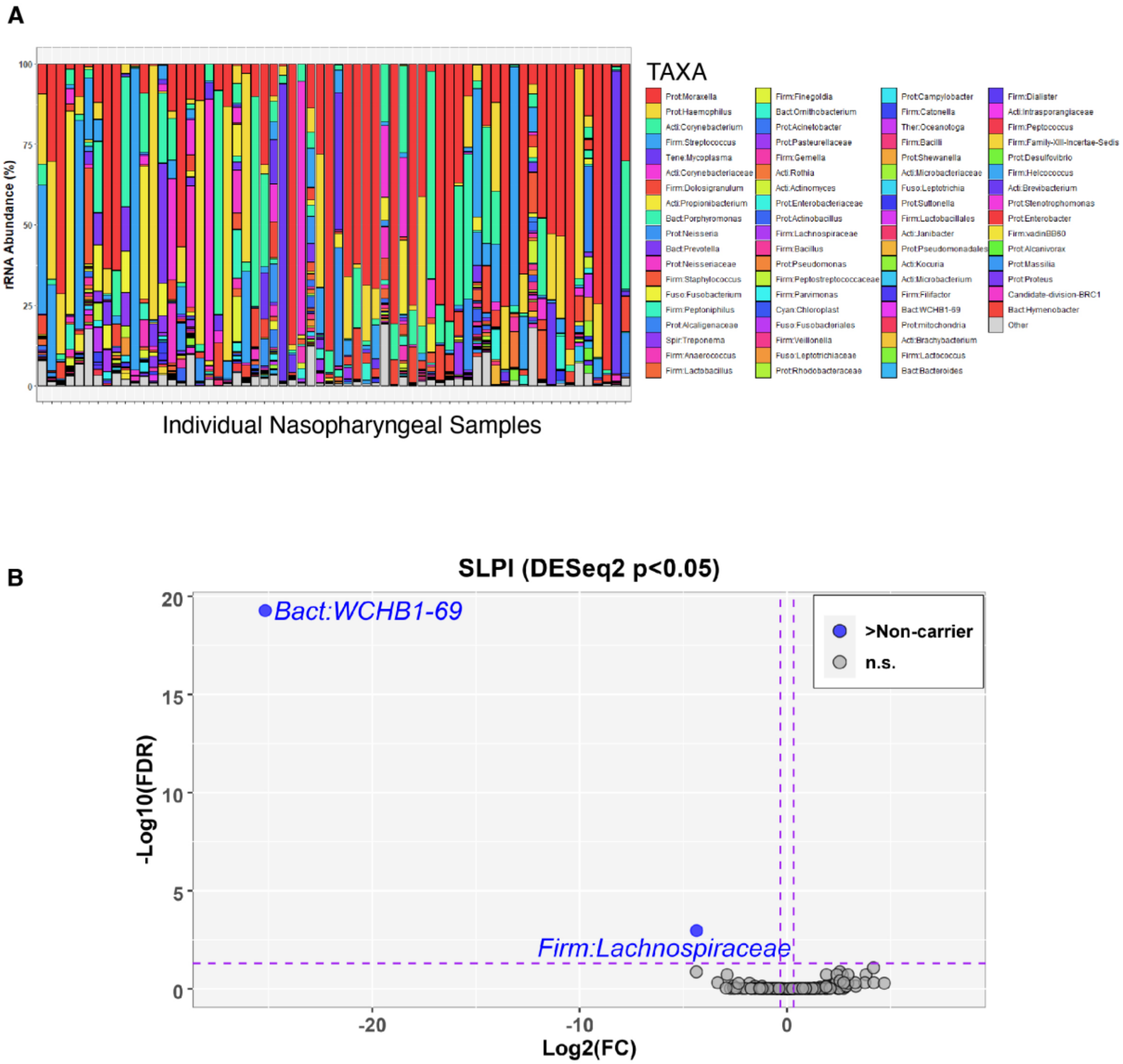

Figure S6. Relative abundance of individual taxa in the nasopharynges of carriers and non-carriers of the *SLPI* c.394+1G>T variant. **(A)** Individual profiles for nasopharyngeal microbiota. **(B)** DESeq2 analysis identified *Lachnospiraceae* (FDR-adjusted-*p* = 0.001) and *WCHB1-69* (FDR-adjusted-*p* = 5.29x10^{-20}) as significantly enriched in non-carriers.

Table S1. Genes moderately co-expressed with *SLPI* in saliva samples of children with OM

**Gene**	**Estimate**	**FDR-adjusted *p***
A2M	0.573841	0.001135
ACIN1	0.648854	0.00014
AGT	0.501901	0.005535
AKR1A1	0.505283	0.005176
AKR1C1	0.505817	0.005121
AKT2	0.503783	0.005333
ALB	0.505817	0.005121
ALDH1A1	0.504172	0.005292
AMBP	0.505006	0.005205
AMOTL2	0.509103	0.004795
APOA1	0.50401	0.005309
APOA2	0.607269	0.000477
APOB	0.50401	0.005309
APOH	0.503709	0.005341
ARL8B	0.612491	0.000413
ARSA	-0.52971	0.003125
ASGR2	0.503048	0.005411
ASPH	0.63529	0.000213
ATP6V1E1	0.558853	0.001626
CALU	0.537509	0.002638
CANX	0.552688	0.001876
CAPRIN1	0.574889	0.001106
CAVIN1	0.509241	0.004781
CCN1	0.589419	0.000767
CCNK	0.521506	0.003717
CCT8	0.562096	0.001507
CD177	0.535941	0.002731
CDC42	0.657704	0.000106
CDK12	0.525264	0.003435
CFL1	0.500884	0.005647
CHMP2B	0.576332	0.001067
CHMP4B	0.597088	0.000627
CLDN1	0.697162	2.64E-05
CLIC1	0.544117	0.002279
CLU	0.502897	0.005427
COL1A1	0.524117	0.003519
COPB2	0.603721	0.000525
COTL1	0.508877	0.004817
COX4I1	0.50488	0.005218
CPPED1	0.610578	0.000435

CRK	0.521461	0.003721
CTSA	0.556926	0.001701
DAD1	0.505188	0.005186
DCTN5	0.584708	0.000865
DDB1	0.578108	0.001021
DDX6	0.519885	0.003845
DHCR24	0.635229	0.000214
DMKN	0.652743	0.000124
DSC3	0.577578	0.001035
DSG3	0.512547	0.004472
DVL3	0.527439	0.00328
DYNLRB1	0.508184	0.004884
EEF1A1P16	0.51365	0.004372
EEF1A1P9	0.521451	0.003721
EIF1	0.518014	0.003997
EIF3A	0.518198	0.003982
EIF3D	0.59548	0.000655
EIF3J	0.530155	0.003095
EIF3L	0.564701	0.001416
EIF4G2	-0.53048	0.003074
EWSR1	0.61709	0.000363
FADS1	0.552047	0.001904
FAM91A1	0.540749	0.002457
FASN	0.599713	0.000585
FBRS	0.697778	2.58E-05
FGA	0.50401	0.005309
FGG	0.503198	0.005395
FKBP1A	0.626625	0.000276
FLNB	0.628649	0.00026
FSCN1	0.505551	0.005148
GABRE	0.509855	0.004723
GADD45A	0.668885	7.28E-05
GANAB	0.529007	0.003172
GAPDHP63	0.541209	0.002432
GIPC1	0.55062	0.001967
GNB2	0.507767	0.004925
GNG10	0.551972	0.001907
GNS	0.568114	0.001305
GREM1	-0.50534	0.00517
GSN	0.527925	0.003246
H2AZ1	0.642698	0.00017
HCFC1	0.500239	0.005719
HDGF	0.541464	0.002418

HMGB1	0.535265	0.002771
HMGB2	0.553306	0.00185
HNRNPD	0.550518	0.001972
HNRNPF	0.520787	0.003773
HNRNPUL1	0.665784	8.09E-05
HNRNPUL2	0.635676	0.000211
HOPX	0.581332	0.000942
HSPD1	0.545244	0.002222
HYOU1	0.684598	4.20E-05
IDH1	0.53994	0.002501
IGF2	0.502202	0.005502
IPO5	0.528593	0.0032
ITGA2	0.5277	0.003262
ITPRID2	0.719767	1.08E-05
KIDINS220	0.525947	0.003385
KIF1B	0.585887	0.000839
LAMC1	0.509411	0.004765
LENG8	0.628098	0.000264
LGALS3BP	0.503952	0.005315
LRRC37A4P	0.505299	0.005174
MACROH2A1	0.603566	0.000528
MAGED1	0.507356	0.004966
MAP4	0.535168	0.002777
MAPRE1	0.504014	0.005308
MCM3	0.511301	0.004586
MGRN1	0.514852	0.004266
MIA2	0.566865	0.001345
MIF	0.506996	0.005002
MLF2	0.522356	0.003652
MT-ATP8	-0.66527	8.23E-05
MT-ND4L	-0.63711	0.000202
MTCH1	0.558742	0.00163
MTCO1P40	-0.53318	0.0029
MTCYBP36	-0.74137	4.21E-06
MTND1P23	-0.62253	0.000311
MTND2P28	-0.54021	0.002486
MYH9	0.507076	0.004994
MYL6	0.506571	0.005045
NFX1	0.511914	0.00453
NME2	0.509074	0.004797
NQO1	0.504855	0.00522
ORM1	0.515496	0.00421
OSTF1	0.521441	0.003722

PCBP1	0.607229	0.000478
PCBP2	0.56838	0.001296
PDIA3	0.520058	0.003831
PERP	0.701355	2.25E-05
PGAM1	0.585301	0.000852
PGK1	0.605051	0.000507
PHB1	0.558504	0.00164
PNISR	0.522215	0.003662
POTEKP	0.513951	0.004345
PPP4R2	0.578942	0.001
PRRC2A	0.561697	0.001521
PSMA7	0.552954	0.001865
PSMB7	0.631568	0.000238
RHOB	0.516269	0.004144
RLIM	0.506791	0.005022
RPL23AP42	0.535762	0.002741
RPL37A	0.510059	0.004703
RPL39	0.510591	0.004653
RPL8	0.555652	0.001752
RPN2	0.513361	0.004398
RPRD2	0.537172	0.002658
RPS21	0.507118	0.00499
RPS23	0.502509	0.005469
RPS27A	0.543926	0.002289
RPS7	0.52245	0.003644
RPS9	0.590437	0.000747
S100A4	0.541364	0.002423
SAR1B	0.627037	0.000273
SARS1	0.539888	0.002504
SDHA	0.518235	0.003979
SEC23B	0.538223	0.002597
SEC63	0.501714	0.005555
SEPTIN9	0.596605	0.000635
SERBP1	0.600884	0.000567
SERINC1	0.570928	0.001219
SERINC5	0.544858	0.002241
SERPINA3	0.57009	0.001244
SERPINE1	0.599006	0.000596
SERPINF2	0.5054	0.005164
SF1	0.521655	0.003706
SH3GLB1	0.507877	0.004914
SIRPA	0.536332	0.002707
SLAIN2	0.501909	0.005534

SLC23A2	0.514479	0.004299
SLC2A1	0.537982	0.002611
SLPI	1	0
SMARCA5	0.52964	0.003129
SNRNP200	0.51568	0.004194
SNX1	0.510294	0.004681
SPARC	0.525856	0.003392
SPTLC2	0.508734	0.00483
SRSF9	0.530965	0.003042
STARD7	0.63485	0.000216
STK40	0.51404	0.004338
STRAP	0.57321	0.001153
STRN	0.543641	0.002303
STT3A	0.507962	0.004906
STT3B	0.588456	0.000786
SURF4	0.561127	0.001541
TIMP2	0.600323	0.000576
TIMP3	0.505099	0.005195
TMEM123	0.542223	0.002377
TMEM33	0.570584	0.001229
TOP1	0.537519	0.002638
TOR1AIP1	0.519045	0.003913
TPI1	0.702265	2.17E-05
TPM1	0.510882	0.004625
TPT1	0.516752	0.004103
TRIM28	0.514993	0.004254
TRPC4AP	0.550019	0.001995
TRRAP	0.520079	0.003829
TSPAN14	0.567849	0.001313
TTYH3	0.502746	0.005443
TUBA1A	0.581935	0.000928
TXN	0.622873	0.000308
U2AF2	0.549956	0.001997
UBA2	0.531431	0.003011
UBE2K	0.542224	0.002377
UGDH	0.558022	0.001658
UGP2	0.506265	0.005076
UPF1	0.528153	0.00323
USO1	0.502938	0.005423
USP22	0.577897	0.001027
WASL	0.56517	0.0014
ZFAND6	0.535663	0.002747
ZNF217	0.518412	0.003964

ZYX	0.552697	0.001876
ZZEF1	0.502757	0.005442

Table S2. Pathways enriched in the protein-protein interaction network connecting *SLPI* with differentially expressed genes

**KEGG Pathway**	**FDR-adjusted *p***
Ubiquitin mediated proteolysis	1.63E-19
TGF-beta signaling pathway	4.81E-13
Pathways in cancer	1.59E-12
HTLV-I infection	1.59E-12
Colorectal cancer	1.65E-11
Hepatitis B	3.80E-10
AGE-RAGE signaling pathway in diabetic complications	5.60E-10
Epstein-Barr virus infection	1.64E-09
Chronic myeloid leukemia	1.72E-09
FoxO signaling pathway	2.21E-09
Cell cycle	2.21E-09
Endocytosis	6.13E-09
Fluid shear stress and atherosclerosis	6.13E-09
Pancreatic cancer	6.13E-09
Thyroid hormone signaling pathway	8.90E-09
Cellular senescence	8.90E-09
Adherens junction	1.59E-08
Endocrine resistance	1.69E-07
Viral carcinogenesis	1.69E-07
Notch signaling pathway	3.03E-07
Kaposi's sarcoma-associated herpesvirus infection	3.03E-07
Chagas disease (American trypanosomiasis)	3.70E-07
Proteoglycans in cancer	1.75E-06
Hepatitis C	3.34E-06
Hedgehog signaling pathway	1.08E-05
Breast cancer	1.49E-05
Osteoclast differentiation	1.65E-05
Signaling pathways regulating pluripotency of stem cells	1.82E-05
IL-17 signaling pathway	2.44E-05
Pertussis	2.73E-05
Hippo signaling pathway	2.94E-05
Spliceosome	3.08E-05
Renal cell carcinoma	3.35E-05
Th17 cell differentiation	4.13E-05
Wnt signaling pathway	4.13E-05
Measles	4.49E-05
Tight junction	4.49E-05
p53 signaling pathway	5.10E-05
T cell receptor signaling pathway	6.20E-05

Mitophagy - animal	6.58E-05
Small cell lung cancer	7.40E-05
RNA transport	7.40E-05
Prostate cancer	0.000127
Aldosterone-regulated sodium reabsorption	0.000144
RIG-I-like receptor signaling pathway	0.000144
Neurotrophin signaling pathway	0.000152
TNF signaling pathway	0.000174
MAPK signaling pathway	0.000181
Bacterial invasion of epithelial cells	0.000255
Shigellosis	0.000256
Toll-like receptor signaling pathway	0.000272
Bladder cancer	0.000325
Focal adhesion	0.000484
Prolactin signaling pathway	0.000533
Thyroid cancer	0.000745
ErbB signaling pathway	0.00107
Transcriptional misregulation in cancer	0.0011
Acute myeloid leukemia	0.00114
Endometrial cancer	0.00139
Epithelial cell signaling in Helicobacter pylori infection	0.00147
NF-kappa B signaling pathway	0.0017
Non-alcoholic fatty liver disease (NAFLD)	0.00243
Pathogenic Escherichia coli infection	0.00346
NOD-like receptor signaling pathway	0.00346
Sphingolipid signaling pathway	0.00357
Salmonella infection	0.00359
Influenza A	0.00364
Non-small cell lung cancer	0.00383
Progesterone-mediated oocyte maturation	0.00431
HIF-1 signaling pathway	0.0047
Fc epsilon RI signaling pathway	0.00475
Leukocyte transendothelial migration	0.00488
Melanogenesis	0.00497
Apoptosis	0.005
Toxoplasmosis	0.00513
VEGF signaling pathway	0.00514
Apelin signaling pathway	0.00518
cAMP signaling pathway	0.00518
Jak-STAT signaling pathway	0.00534
GnRH signaling pathway	0.00624
Amyotrophic lateral sclerosis (ALS)	0.00626
mTOR signaling pathway	0.0066

PI3K-Akt signaling pathway	0.00687
Longevity regulating pathway - multiple species	0.00687
Platinum drug resistance	0.0072
Huntington's disease	0.00727
Amoebiasis	0.00769
Glioma	0.00867
Prion diseases	0.00891
Choline metabolism in cancer	0.00982
Estrogen signaling pathway	0.0122
Chemokine signaling pathway	0.0126
MicroRNAs in cancer	0.0126
Protein processing in endoplasmic reticulum	0.0132
Tuberculosis	0.014
B cell receptor signaling pathway	0.0166
Leishmaniasis	0.0222
Cytosolic DNA-sensing pathway	0.0223
Basal cell carcinoma	0.0223
Insulin signaling pathway	0.0249
Regulation of lipolysis in adipocytes	0.0302
Rheumatoid arthritis	0.0328
EGFR tyrosine kinase inhibitor resistance	0.0328
Th1 and Th2 cell differentiation	0.0351
AMPK signaling pathway	0.0422
Viral myocarditis	0.0434

Table S3. scRNA-seq metrics for control versus 6-hour infected mouse middle ears

**Metric**	**At 0 hour prior to infection**	**At 6 hours post-infection**
Number of cells	2,818	2,557
Reads per cell	18,886	30,252
Genes per cell	1,637	816
Total genes detected	17,719	16,791
Unique molecular identifiers per cell	3,933	3,914
Reads mapped to genome	89%	84%
Reads mapped to transcriptome	65%	68%
